# Small Molecule Inhibitor C188-9 Synergistically Enhances the Demethylated Activity of Low-Dose 5-Aza-2′-Deoxycytidine Against Pancreatic Cancer

**DOI:** 10.3389/fonc.2020.00612

**Published:** 2020-05-08

**Authors:** Rui Kong, Guangming Sun, Xina Li, Linfeng Wu, Le Li, Yilong Li, Fei Wang, Ping Xuan, Shifeng Yang, Bei Sun, Jisheng Hu

**Affiliations:** ^1^Department of Pancreatic and Biliary Surgery, First Affiliated Hospital of Harbin Medical University, Harbin Medical University, Harbin, China; ^2^Key Laboratory of Hepatosplenic Surgery, Ministry of Education, Harbin, China; ^3^School of Computer Science and Technology, Heilongjiang University, Harbin, China

**Keywords:** 5-aza-2′-deoxycytidine, C188-9, STAT3, RASSF1A, DNA methylation, epithelial-to-mesenchymal transition

## Abstract

Aberrant DNA methylation, especially hypermethylation of tumor suppressor genes, has been associated with many cancers' progression. 5-Aza-2′-deoxycytidine (DAC) can reverse hypermethylation-induced gene silencing via regulating DNA methyltransferases (DNMTs) activity, In addition, low-dose of DAC was proved to exert durable antitumor effects against solid tumor cells. Nevertheless, no clinical effect of DAC has been made when fighting against pancreatic cancer. Hence, it is necessary to raise a novel therapeutic strategy that further enhance the efficacy of DAC but not increase side effect, which impede the utilization of DAC. In the present study, we have discovered that C188-9, a novel signal transduction activator of transcription (STAT) inhibitor, could improve the antitumor effects of low-dose DAC *in vivo* and *in vitro*. Further study demonstrated that such improvement was attributed to re-expression of Ras association domain family member 1A (RASSF1A), a well-known tumor suppressor gene. Bisulfite sequencing PCR (BSP) assay showed that C188-9 combined with DAC treatment could significantly reverse the hypermethylation status of RASSF1A promoter, which indicated that C188-9 could enhance the demethylation efficacy of DAC. Our data demonstrated that DNA methyltransferase 1 (DNMT1) was the underlying mechanism that C188-9 regulates the demethylation efficacy of DAC. Overall, these findings provide a novel therapeutic strategy combining low-dose DAC and C188-9 to improve therapeutic efficacy by inhibiting DNMT1-inducing promoter methylation.

## Introduction

Pancreatic ductal adenocarcinoma is highly lethal, for which the 5-year relative survival is currently 8% (1), and it is predicted to become the second leading cause of cancer-related deaths by 2030 in the United States (2). The low survival rate is mainly attributed to the late stage at which most patients are diagnosed. More than 80% of patients are diagnosed with local advanced or distant metastasis (1), which leads to loss of chance for surgery and poor prognosis.

Epigenetically silenced tumor-suppressing genes by promoter methylation are recently recognized as a major contributor to cancer progression. Therefore, DNA-demethylating agents are emerging as a novel approach to cancer therapy. 5-Aza-2′-deoxycytidine, also known as decitabine (DAC), can reactivate the expression of genes silenced by DNA methyltransferases (DNMTs) activity, and it has shown a dramatically therapeutic benefit in hematological malignancies (3). Besides, the clinical effectiveness of DAC therapy in solid tumors has been demonstrated in recent years (4, 5); unfortunately, most of the clinical trials investigating DAC alone or in combination with other agents have shown relatively disappointing results (6). Moreover, myelosuppression, as the major toxicity of DAC, seriously restricted its high-dose application in malignancy. However, low-dose DAC was proved to be relatively well tolerated in both hematological malignancies and solid tumors. Although pancreatic cancer cells are sensitive to monotherapy of DAC, there were limited evidence for the optimal strategy of appropriate doses and combination therapies with DAC. Hence, a novel therapeutic strategy that reduces toxicity while enhancing demethylation efficiency of DAC is essential.

There are seven members of the signal transduction activator of transcription (STAT) family that regulate responses to extracellular signals (7), and STAT3 is one of the best-studied STAT members owing to its crucial roles in various physiological processes. Aberrant STAT3 pathway activation has been observed in many types of cancer and also functions as an oncogene in promoting cell proliferation, metastasis, epithelial–mesenchymal transition (EMT) and stemness, and chemotherapy resistance (8). Hence, various drugs targeting STAT3 have been discovered, including decoy oligonucleotides, peptidomimetics, and inhibitors targeting the Src homology 2 (SH2) domain (9). STAT3 has also been proven to induce epigenetic silencing of SHP-1 in malignant T lymphocytes (10). We wondered if inhibitors targeting STAT3 enhance demethylation activity of DAC in pancreatic ductal adenocarcinomas (PDACs). C188-9, a new and effective STAT3 inhibitor targeting SH2 domain, was first reported to inhibit granulocyte-colony stimulating factor (G-CSF)-induced Stat3 phosphorylation and induce apoptosis of acute myeloid leukemia (AML) cell lines and primary samples (11). This STAT3 inhibitor showed antitumor activity in hepatocellular carcinoma (12), head and neck squamous cell carcinoma (13), and nonsmall cell lung cancer (14).

The aim of the present study was to explore a new strategy that enhances the antitumor effect of low-dose DAC against pancreatic cancer. Using an orthotopic tumor model and other experiments in *vitro*, we found that treatment with low-dose DAC in conjunction with C188-9 got better antitumor effects than either monotherapy, especially high-dose DAC, without inducing more severe side effect. Such increase in efficacy owes to further re-expression of Ras association domain family member 1A (RASSF1A). Additionally, it is demonstrated that DNA methyltransferase 1 (DNMT1) was responsible for the C188-9 inducing the demethylation activity of DAC.

## Materials and Methods

### Cell Lines and Reagents

The human pancreatic cancer cell lines BxPC-3 and PANC-1 were purchased from the American Type Culture Collection. BxPC-3 and PANC-1 cell lines were routinely cultured in Roswell Park Memorial Institute (RPMI) 1640 medium (HyClone, USA) supplemented with 10% fetal bovine serum (Gibco, USA), penicillin (100 U/ml), and streptomycin (100 mg/ml). All cells were cultured at 37°C with 5% CO_2_. DAC (Sigma-Aldrich, USA) and C188-9 (Selleck, USA) were dissolved in dimethyl sulfoxide (DMSO, Sigma) and stored at −20°C. Antibodies used in this study included antibodies against E-cadherin (Cell Signaling Technology, Inc., MA, USA), β-actin, (Santa Cruz Biotechnology, CA, USA), RASSF1A, N-cadherin, (Abcam Inc., MA, USA), Snail1, DNMT1, and Vimentin (ProteinTech Group Inc., IL, USA).

### Bisulfite Sequencing PCR Assay

Genomic DNA was isolated from pancreatic cancer cells with or without treatment using genomic DNA extraction kit according to the manufacturer's instructions [SK8224, Sangon Biotech (Shanghai) Co., Ltd.], which was then sequenced by sodium bisulfite treatment. Sequencing primers were as follows: 5′-TTYGTTTGTTTTTTTYGTTAGG-3′ (forward) and 5′-ACAACCAATCAAAAATAACAACR-3′ (reverse). PCR cycle conditions were as follows: 98°C × 4 min for 1 cycle; 20 cycles × (94°C × 45 s, 66°C × 45 s, 72°C × 1 min), 20 cycles × (94°C × 45 s, 56°C × 45 s, 72°C × 1 min); 72°C 8 min for 1 cycle. PCR products were gel purified and cloned into pUC18-T vectors. Ten clones from each sample were selected for sequencing.

### Clonogenic Survival of Cancer Cells

Pancreatic cancer cells were seeded at a density of 1–2 × 10^4^ cells/well in 6-well plates and treated with DAC with or without C188-9. The cells were cultured for 10 days. The culture medium or the same medium containing treatment agents were replaced every 3 days. After fixing with methanol and staining with 1% crystal violet, the colonies were photographed (RX100, SONY, Japan) and counted manually.

### Wound Healing Assay

Wound healing assay was performed as described previously (15). Briefly, cells grown onto 6-well plates were pretreated with mitomycin C (10 μg/ml) 2 h before an artificial “wound” was created with a 200-μl pipette tip at 0 h and then incubated in serum-free medium for PANC-1 or 1% serum medium for BxPC-3. Photographs were taken at 0 and 24 h with a 10× Olympus microscope. The percentage of wound closure was estimated by Image J software.

### Transwell Assay

Transwell assay was performed as described previously (15–17). The motility and invasiveness of cells exposed to DAC with or without C188-9 was assayed using 8-μm pore size Falcon® inserts coated with or without Matrigel (BD, USA). Cells exposed to indicated treatment for 48 h were collected, and 5 × 10^5^ cells in 200 μl of serum-free medium were placed in the upper part of the Transwell unit and allowed to invade for 24 h. The lower part of the Transwell unit was filled with 500 μl medium containing 10% fetal bovine serum (FBS). After incubation, noninvasive cells on the upper part of the membrane were removed with a cotton swab. Invasive cells on the bottom surface of the membrane were fixed in methanol and then stained with crystal violet. The number of cells in five randomly selected fields (20×) was counted; all assays were performed in triplicate.

### Western Blotting

The methodology has been described previously (15, 18–20). In brief, pancreatic tissues or cells were sonicated in radioimmunoprecipitation assay (RIPA) buffer (Beyotime Institute of Biotechnology, Beijing, China) that contained protease inhibitor cocktail (Sigma, USA) and phosphatase inhibitor (Roche, Shanghai, China) and homogenized. The samples containing 20–40 μg protein were electrophoresed on 10% polyacrylamide sodium dodecyl sulfate (SDS) gels and transferred to polyvinylidene difluoride (PVDF) membranes. The membranes were blocked with 5% skim milk prior to incubation with primary antibodies and subsequently incubated with horseradish peroxidase-conjugated secondary antibody (Santa Cruz Biotech, Santa Cruz, CA, USA). Protein bands were visualized with the enhanced chemiluminescence kits (Pierce Chemical, Rockford, IL, USA). β-Actin was simultaneously determined as a loading control, and the level of protein expression was calibrated as the relative band density to that of β-actin.

### RNA Isolation, Reverse Transcription, and Quantitative Real-Time PCR

Total RNA was extracted and isolated using a AxyPrep Multisource Total RNA Miniprep Kit (Axygen Biosciences, USA) according to the manufacturer's instruction; the first-strand complementary DNA (cDNA) was synthesized by the ReverTra Ace qPCR RT Master Mix (Toyobo Co. Ltd.) according to the manufacturer's instruction. Quantitative real-time PCR (qRT-PCR) was conducted as previously described (15, 17). Briefly, qRT-PCR (SYBR Green Assay, Roche Diagnostics GmbH) was performed on 7500 FAST Real-Time PCR System (Applied Biosystems). The relative expression levels of messenger RNAs (mRNAs) were calculated and quantified using the 2^−ΔΔ^T method after normalization for the expression of the control, and glyceraldehyde 3-phosphate dehydrogenase (GAPDH) served as the endogenous control. The primer sequences were designed by Primer 5.0 and purchased from Invitrogen. The primer sequences are described in Table S1.

### Transfection

Small-interfering RNA (SiRNA) against RASSF1A was purchased from RIBOBIO (Guangzhou, China), and DNMT1 (NM_001379) overexpression plasmid was purchased from Genechem (Shanghai, China). For transient transfections, BxPC-3 and PANC-1 cells were cultured for 24 h in 6-well plates with 50 nM siRNA or 2 μg of DNA and 4 μl of Lipofectamine 2000 (Invitrogen Life Technologies) according to the manufacturer's recommendations. The target sequences of siRNAs are listed in Table S2.

### Animal Experiments

Five-week-old female nude mice were obtained from Beijing Vital River Laboratory Animal Technology. All surgical procedures and care administered to the animals have been approved and reviewed by the Animal Care and Use Committee of The First Clinical Medical School of Harbin Medical University (Harbin, China). Orthotopic tumor models were created as previously described (17). Briefly, tumors were established by subcutaneous injection of 5 × 10^6^ BxPC-3-Luc cells into the dorsal flank of the mice. When the volume of tumors increased to ~120 mm^3^, 1-mm^3^ pieces of the tumor were translocated into mice's pancreatic tails, respectively. The animals were imaged weekly by NightOWL II LB983 *in vivo* imaging system (Berthold Technologies GmbH & Co. KG, Germany). After 10 days, the host mice were randomly assigned to five groups: (1) control group (equal volume of DMSO), (2) 0.125 DAC group (0.125 mg/kg, administered once daily by i.p. injection), (3) 1 DAC group (1 mg/kg, administered once daily by i.p. injection), (4) C188-9 group (100 mg/kg, administered once daily by i.p. injection), (5) DAC plus C188-9 group (0.125 mg/kg for DAC and 100 mg/kg for C188-9). After for 4 weeks treatment, the mice were euthanized. After sacrifice, blood was collected for white blood cell (WBC) counts, and the tumor was fixed in 4% formaldehyde or stored in −80°C for further analysis.

### Immunohistochemical Staining

The immunohistochemical staining protocol has been described previously (15, 21, 22). In brief, paraffin-embedded tissue sections (5 μm) were immunostained with anti-RASSF1A, anti-E-cadherin, anti-N-cadherin, anti-Vimentin, anti-ki-67, anti-Snail1, and anti-DNMT1. The number of positive cells was counted in five randomly selected microscopic fields (10×, Olympus, Japan).

### Statistical Analysis

Statistical analysis was performed with SPSS 19.0 software (IBM, USA). Results were expressed as the mean ± standard deviation (SD). Additionally, the continuous data were analyzed by ANOVA test and Student's *t*-test. The differences were considered statistically significant when ^*^P < 0.05, ^**^P < 0.01, and ^***^P < 0.001.

## Results

### C188-9 Increases DAC Efficacy in Inhibiting Proliferation of Pancreatic Cancer Cells *in vitro* and *in vivo*

**Figure 1:**
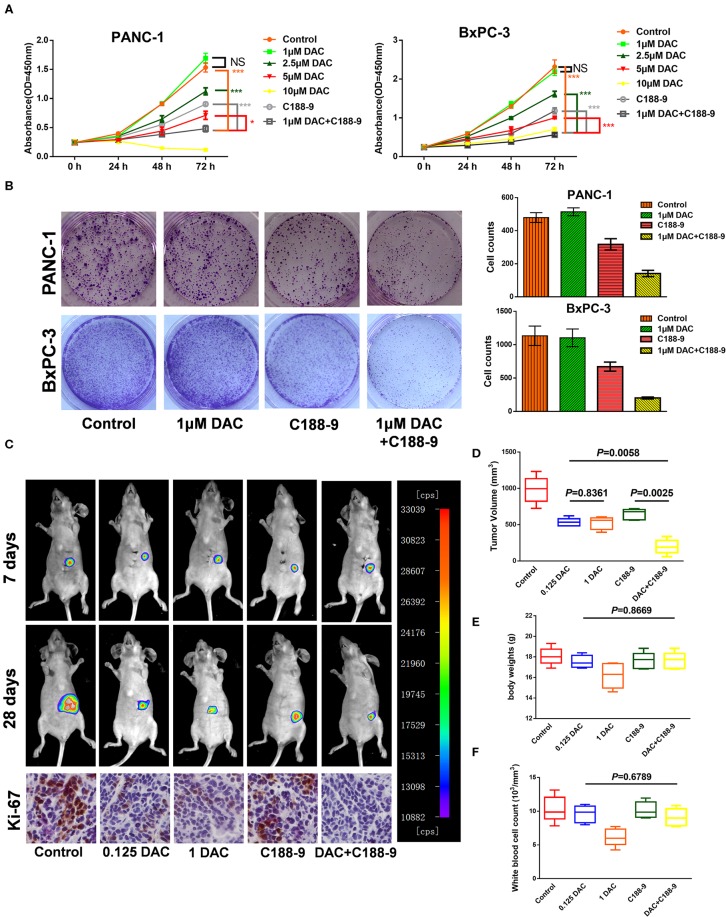
C188-9 increases 5-aza-2′-deoxycytidine (DAC) efficacy in inhibiting proliferation of pancreatic cancer cells *in vitro* and *in vivo*. **(A)** Cell growth curves of PANC-1 and BxPC-3 cells under indicated treatment using Cell Counting Kit-8 (CCK-8) assay. **(B)** Clonogenic death of PANC-1 cells and BxPC-3 cells under 1 μM DAC treatment with or without 10 μM C188-9 treatment. **(C)** Representative bioluminescence imaging of orthotopic xenograft mice at the days 7 and 28 (upper two panel). Representative images of immunohistochemistry analyses to detect Ki-67 in orthotopic tumors from five indicated treatment group (lower panel). **(D)** Tumor volume of primary tumors removed from orthotopic xenograft mice after sacrifice at day 28. **(E)** Body weight of five group orthotopic xenograft mice after treatment for 28 days. **(F)** After sacrifice, blood was collected for white blood cell counts. The statistical significance between different groups was calculated with Student's *t*-test. Data are presented as mean values ± SD. The statistical significance between different groups was calculated with Student's *t*-test. Data are shown as the mean ± SD of three replicates; *P < 0.05; ***P < 0.001; NS, not significant.

In order to study the effect of C188-9 in enhancing sensitivity of pancreatic cancer cells to low-dose DAC treatment, Cell Counting Kit-8 (CCK-8) assay and clonogenic assay were performed to test the effect of different doses of DAC in growth of pancreatic cancer cells *in vitro*. Through CCK-8 assay, we found that 1 μM DAC treatment for 72 h cannot significantly suppress proliferation of pancreatic cancer cells (Figure 1A), which is consistent with previous study (23), while after combination treatment with 10 μM C188-9, 1 μM DAC treatment showed significant suppression of proliferation in both BxPC-3 and PANC-1 cell lines. Moreover, the combination treatment was even significantly superior to 2.5 μM, 5 μM DAC treatment, or 10 μM C188-9 treatment alone (Figure 1A). In addition, combining C188-9 treatment with low-dose DAC significantly inhibited colony formation of pancreatic cancer cells (Figure 1B). Based on *in vitro* data, *in vivo* assay was also performed to test the synergistic antitumor effect of combined treatment and whether such combined treatment induced more severe side effects compared to monotherapy. Hence, an orthotopic pancreatic cancer model with BxPC-3-Luc cell line was also introduced to determine whether combination treatment of C188-9 and DAC inhibits proliferation of pancreatic cancer cells *in vivo*. Through *in vivo* imaging system and orthotopic tumor size after sacrifice, we found that the suppression effect of monotherapy with low-dose DAC (0.125 mg/kg) was not inferior to that of the treatment with high-dose DAC (1 mg/kg), and combined treatment with C188-9 and low-dose DAC significantly suppresses proliferation of orthotopic tumor compared to monotherapy with DAC or C188-9 (Figures 1C,D) without influencing body weight and WBC counts (Figures 1E,F). Moreover, tumor cell proliferation was assessed using immunohistochemistry for the Ki-67 protein. Different doses of DAC results in the reduction in proliferation rate, and it continues to decrease when combined with C188-9 (Figure 1C, inferior panel). These data demonstrated that low dose of DAC could be an effective therapy against proliferation of pancreatic cancer cells *in vivo* and not inferior to the treatment with a high dose of same agent, and C188-9 could effectively enhance the efficacy of DAC *in vitro* and *in vivo*.

### C188-9 Increases DAC Efficacy in Inhibiting Migration, Invasion, and EMT of Pancreatic Cancer Cells *in vitro* and *in vivo*

**Figure 2:**
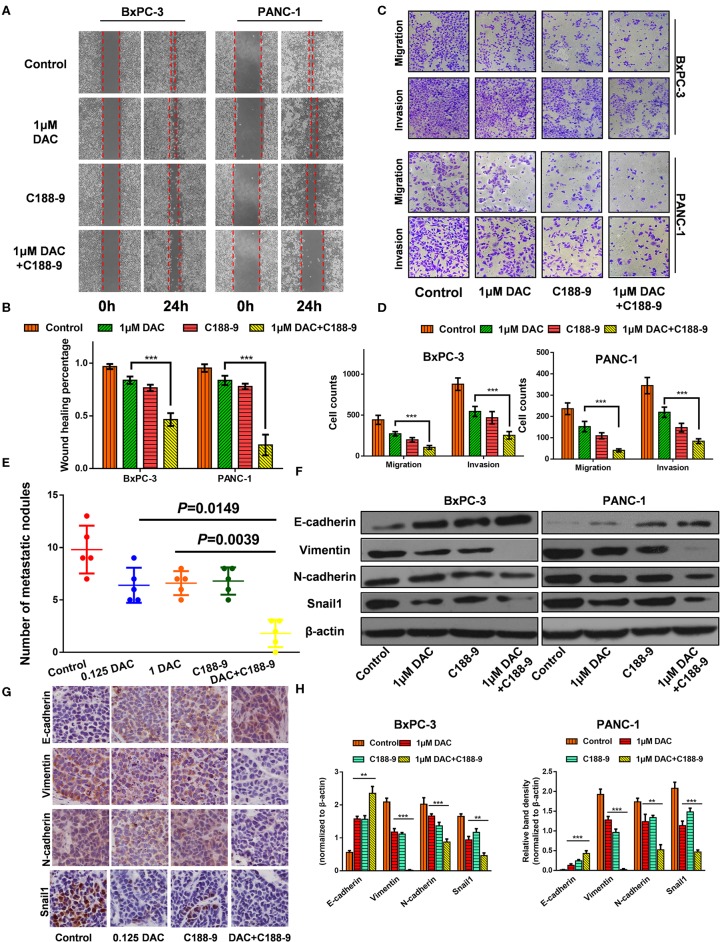
C188-9 increases 5-aza-2′-deoxycytidine (DAC) efficacy in inhibiting migration, invasion, and epithelial–mesenchymal transition (EMT) of pancreatic cancer cells *in vitro* and *in vivo*. **(A,B)** Wound healing assay and **(C,D)** Transwell assay of cells treated with 1 μM DAC and/or 10 μM C188-9 for 48 h. **(E)** Metastatic nodes of all five groups of orthotopic xenograft mice model were calculated. **(F,H)** Vimentin, N-cadherin, E-cadherin, and Snail1 were detected by Western blot; β-actin served as the internal control. **(G)** Vimentin, N-cadherin, E-cadherin, and Snail1 were detected by immunohistochemistry in the control group, 0.125 DAC group, C188-9 group, and DAC plus C188-9 group of orthotopic tumor model. The statistical significance between different groups was calculated with Student's *t*-test. Data are shown as the mean ± SD of three replicates; **P < 0.01; ***P < 0.001.

We also tested to determine whether C188-9 acts to enhance efficacy of DAC in inhibiting the migration and invasion of pancreatic cancer cells. After exposure to indicated treatment, pancreatic cancer cell lines of BxPC-3 and PANC-1 were utilized for Transwell and wound healing assay. The results showed that C188-9 enhanced DAC-induced suppression of migration and invasion of pancreatic cancer cells (Figures 2A–D). The orthotopic pancreatic tumor model was also constructed to verify whether such combination treatment would achieve synergetic effect in inhibiting forming metastatic nodes. In the *in vivo* assay, significantly fewer visible metastasis nodes were found in the combination treatment group compared with the control group, low-dose DAC group, high-dose DAC group, and C188-9 group (Figure 2E). All the data above demonstrated that combined treatment with C188-9 and low-dose DAC exhibited synergetic effect in suppressing migration and invasion of pancreatic cancer cells *in vivo* and *in vitro*.

EMT plays a key role in regulating motility and invasiveness of cancer cells; epithelial and mesenchymal markers were examined by Western blot and immunohistochemistry. Western blot showed that DAC alone or in combination with C188-9 significantly weakened the expression of Vimentin, N-cadherin, and Snail1 and increased the expression of E-cadherin (Figures 2F,H). As shown in Figure 2G, immunohistochemical assays are consistent with the data obtained from the Western blotting experiments. Collectively, our results suggest that C188-9 can augment the antimetastasis effect of DAC by inhibiting EMT.

### RASSF1A Was Involved in DAC-Induced Inhibition of Proliferation and EMT Process

**Figure 3:**
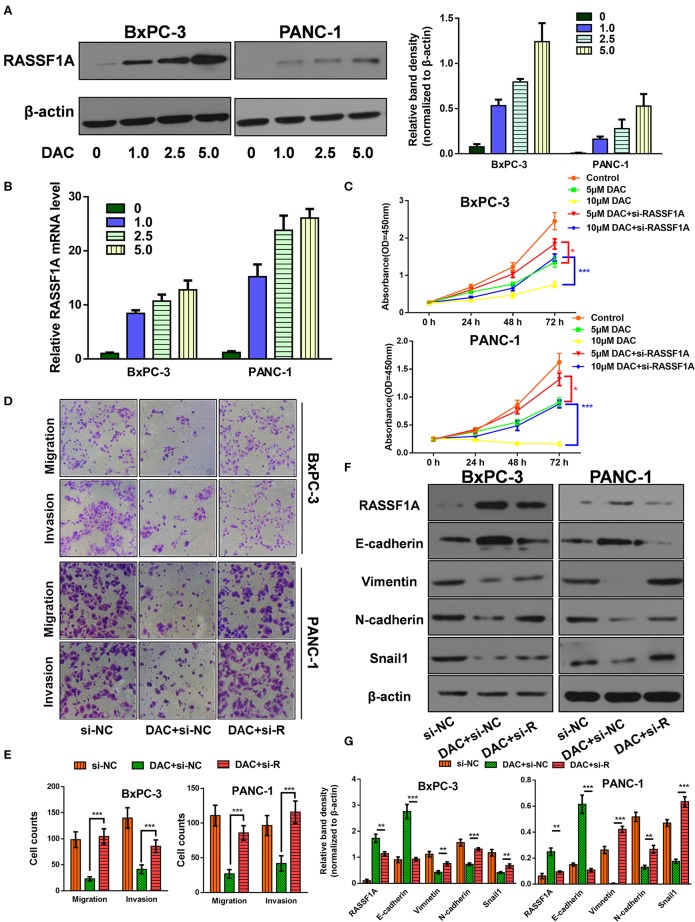
Ras association domain family member 1A (RASSF1A) was involved in 5-aza-2′-deoxycytidine (DAC)-induced inhibition of proliferation and epithelial–mesenchymal transition (EMT) process. **(A)** Immunoblot for the expression level of RASSF1A in pancreatic cancer cells following treatment with incremental concentration of DAC (1, 2.5, 5 μM). **(B)** Level of RASSF1A messenger RNA (mRNA) in pancreatic cancer cells following treatment with incremental concentration of DAC (1, 2.5, 5 μM) were tested by quantitative real-time PCR (qRT-PCR). **(C)** Cell growth curves of PANC-1 and BxPC-3 under 5 μM DAC treatment with or without RASSF1A knockdown. **(D,E)** Migration and invasion of PANC-1 and BxPC-3 cells under 5 μM DAC treatment with or without RASSF1A knockdown were tested by Transwell assay. **(F,G)** RASSF1A, Vimentin, N-cadherin, E-cadherin, and Snail1 of PANC-1 and BxPC-3 cells under 5 μM DAC treatment with or without RASSF1A knockdown were detected by Western blot; β-actin served as the internal control. The statistical significance between different groups was calculated with Student's *t*-test. Data are shown as the mean ± SD of three replicates; *P < 0.05; **P < 0.01; ***P < 0.001. si-R, si-RASSF1A.

The Ras association domain family member 1A (RASSF1A) is one of the most frequently reported tumor suppressor genes that is inactivated by promoter hypermethylation in PDAC (24). Hence, it is reasonable to assume that DAC could help restore its transcription and expression in pancreatic cancer cells, which is consistent with previous studies (24, 25) and our results (Figures 3A,B). Then, to determine the role of RASSF1A in DAC-mediated inhibition of proliferation and EMT *in vitro*, siRNA against RASSF1A was introduced. As shown in the CCK-8 assay (Figure 3C), RASSF1A knockdown significantly attenuated DAC-induced death of pancreatic cancer cells. Moreover, silencing of RASSF1A helps restore the capability of motility, invasion, and EMT of pancreatic cancer cells (Figures 3D–G). Hence, we can conclude that RASSF1A, as the target of DAC treatment, participates in DAC-induced inhibition of proliferation and EMT process.

### C188-9 Increases DAC Efficacy in Re-expression of RASSF1A by Reversing the Hypermethylation Status of Its Promoter CpG Island

**Figure 4:**
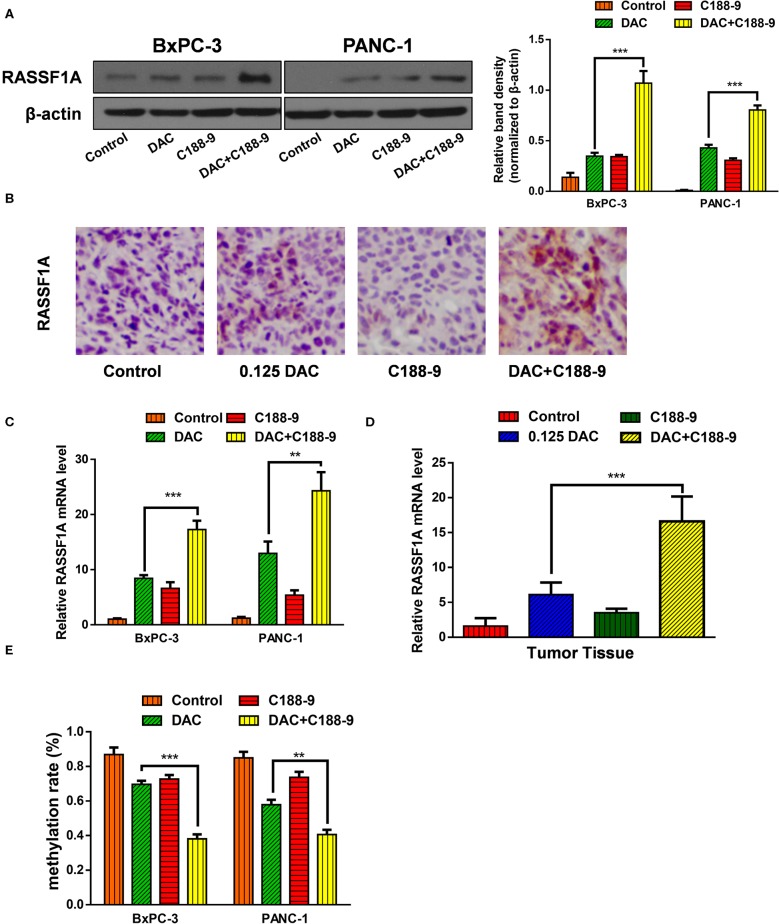
C188-9 increases 5-aza-2′-deoxycytidine (DAC) efficacy in re-expression of Ras association domain family member 1A (RASSF1A) by reversing the hypermethylation status of its promoter CpG island. **(A)** Immunoblot for the expression level of RASSF1A in pancreatic cancer cells following 1 μM DAC treatment with or without 10 μM C188-9. **(B)** RASSF1A levels were detected by immunohistochemistry in the control group, 0.125 DAC group, C188-9 group, and DAC plus C188-9 group of orthotopic tumor model. **(C,D)** Level of RASSF1A mRNA were detected by quantitative real-time PCR (qRT-PCR) using cancer cell **(C)** and tumor tissue **(D)**. **(E)** Methylation rate of RASSF1A promoter in pancreatic cancer cells under 1 μM DAC treatment with or without 10 μM C188-9 by bisulfite sequencing PCR (BSP) assay. The statistical significance between different groups was calculated with Student's *t*-test. Data are shown as the mean ± SD of three replicates; **P < 0.01; ***P < 0.001.

To further ascertain whether C188-9 enhances the efficacy of DAC by regulating methylation status of promoter, based on the previous data that RASSF1A participates in DAC-induced function, we chose RASSF1A as target of further experiment. First, we tested whether combined treatment significantly increased the protein expression of RASSF1A. Both the Western blot assay and immunohistochemistry assay suggested that combined treatment could significantly increase protein level of RASSF1A (Figures 4A,B). Furthermore, the mRNA level of RASSF1A of pancreatic cancer cells or tumor tissue was also tested when exposed to DAC treatment combined with or without C188-9 and obtained consistent results (Figures 4C,D), which suggested that combined treatment could restore RASSF1A expression at transcription level. As methylation status of promoters is crucial for transcriptional activity, the methylation status of RASSF1A promoter CpG island was also tested by bisulfite sequencing PCR (BSP) assay. As shown in Figure 4E, compared to the DAC alone group, the methylation rate of RASSF1A promoter CpG island was significantly decreased when treated with DAC and C188-9. Hence, we presume that C188-9 could enhance the demethylation activity of DAC, at least when targeting RASSF1A.

### C188-9 Enhance the Demethylation Activity of DAC by Targeting DNMT1

**Figure 5:**
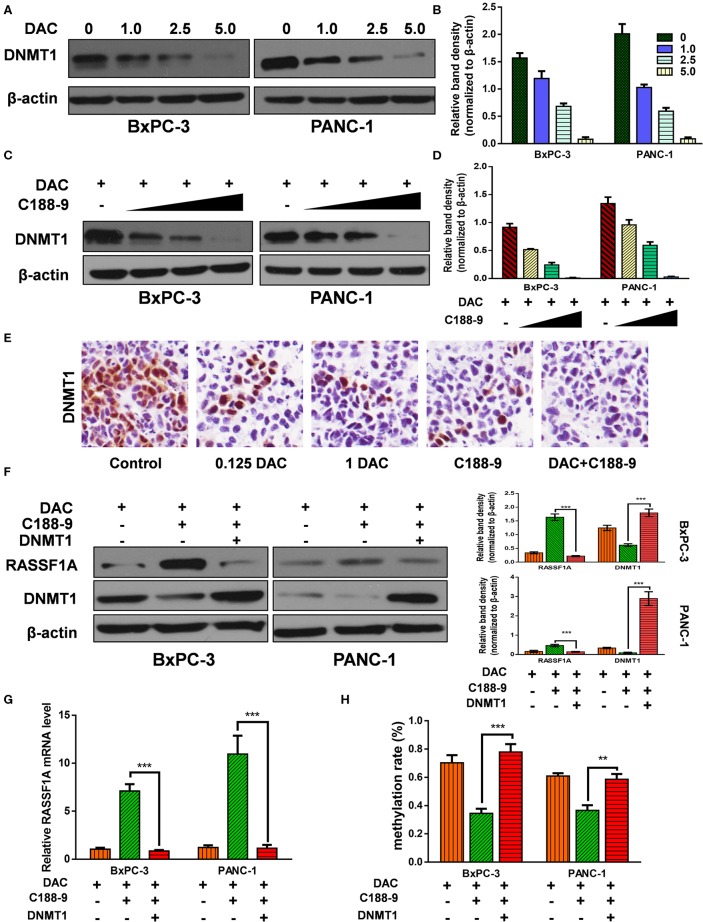
C188-9 enhance the demethylation activity of 5-aza-2′-deoxycytidine (DAC) by targeting DNA methyltransferase 1–signal transducer and activator of transcription 3 (DNMT1–STAT3) interaction. **(A,B)** Immunoblot for the expression level of Ras association domain family member 1A (RASSF1A) in pancreatic cancer cells following treatment with incremental concentration of DAC (1, 2.5, 5 μM). **(C,D)** RASSF1A level was detected with incremental concentration of C188-9 (10, 25, 50 μM) under DAC treatment. **(E)** DNMT1 level were detected by immunohistochemistry in the control group, 0.125 DAC group, 1 DAC group, C188-9 group, and DAC plus C188-9 group of orthotopic tumor model. **(F)** BxPC-3 and PANC-1 cells were, respectively, under DAC treatment, DAC plus C188-9 treatment, and combined treatment plus DNMT overexpression; the cell lysates were subjected to Western blot to test protein level of RASSF1A and DNMT1. Three groups above were also subjected to **(G)** quantitative real-time PCR (qRT-PCR) to text the RASSF1A mRNA level and **(H)** to BSP assay to detect methylation status of RASSF1A promoter. The statistical significance between different groups was calculated with Student's *t*-test. Data are shown as the mean ± SD of three replicates; **P < 0.01; ***P < 0.001.

After verifying that C188-9 promotes DAC-induced effect by reversing hypermethylation status of RASSF1A promoter, next, we tried to explore the mechanism how C188-9 regulated the demethylation capacity of DAC. DNMT1 is mainly responsible for maintaining methylation status of CpG islands (26), and DAC, functioning as DNMT inhibitor, can induce depletion of DNMT1, which is also consistent with our results (Figures 5A,B). Moreover, we also tried to demonstrate whether C188-9, under DAC treatment, could further decrease the expression of DNMT1. As shown in Figures 5C,D, C188-9 significantly decreased DNMT1 expression in a dose-dependent manner under DAC treatment. *In vivo* experiment acquired consistent results (Figure 5E). Thus, we speculated that DNMT1 might be the potential target by which C188-9 enhanced the demethylation activity of DAC. DNMT1-overexpression plasmid was introduced to verify the role of DNMT1 in regulating C188-9-induced increase in demethylation activity of DAC. We first examined the expression of RASSF1A at mRNA and protein level, respectively. As shown in Figures 5F,G, DNMT1 overexpression abolished the increase in RASSF1A expression at mRNA and protein level significantly. Moreover, the methylation rate of CpG island on RASSF1A promoter was also significantly restored when DNMT1 was overexpressed in combined treatment (Figure 5H). Hence, we suppose that DNMT1 might be the target through which C188-9 enhances the demethylation activity of DAC.

## Discussion

Cancer used to be known as a set of diseases driven by genetic alterations, which include mutations in oncogenes and tumor-suppressor genes (27). However, epigenetic processes have been revealed to have an emerging role in cancer development and progression that do not involve changes in the DNA sequence (28, 29). In the last decades, great attention has been paid to epigenetic mechanisms in all kinds of cancer, especially aberrant DNA methylation, which regulates chromatin compaction and repression of gene expression. The reversible nature of epigenetic changes makes them potential therapeutic targets. DAC, a DNMT inhibitor, could reverse hypermethylation-induced gene silencing by forming irreversible covalent bonds with the active sites of DNMT. Even though approved by the Food and Drug Administration (FDA) for the treatment of hematological malignancies, few substantial progress has been made for DAC in the treatment of solid tumor. Recent studies have shown that nanomolar doses of DAC exhibited durable antitumor effects and gene re-expression of solid tumor cells without inducing immediate cytotoxicity (30), which makes low-dose DAC a promising chemotherapy regimen. To further enhance the efficacy of demethylation activity, combined treatment might be a main mode.

Preclinical data have confirmed that STAT3 signaling pathway is pivotal in the development of many human cancers including pancreatic cancer. Hyperactivated STAT3 promotes the growth of pancreatic cancer (31), metastasis, initiation (32), drug resistance, and remodeling of the tumor microenvironment and is associated with patient survival (33). Thus, targeted inhibition of STAT3 could be a novel therapeutic agent applied in clinic to fight against pancreatic cancer. At present, several inhibitors targeting STAT3 have now reached clinical trials (34), such as AZD9150, C188-9, OPB-31121, and OPB-51602. C188-9, based on the scaffold of C188, was identified to bind SH2 domain of STAT3 with high affinity and was proved to function as novel targeted therapy by targeting STAT3. Hence, we aimed to find out whether C188-9 combined with DAC could gain a better antitumor effect without increasing cytotoxicity. The present study provides preclinical evidence that C188-9 increases DAC efficacy in inhibiting proliferation, migration, invasion, and EMT of pancreatic cancer cells *in vitro* and *in vivo*. Moreover, consistent with previous studies, our experiment *in vivo* suggested that low-dose DAC treatment could significantly suppress proliferation of orthotopic tumor and was not inferior to that of the treatment with high-dose DAC. In addition, low-dose of DAC combined with C188-9 did not induce weight reduction or augment of hematological toxicity or decrease. Hence, we assume that C188-9 combined with low-dose DAC might be an effective and safe treatment.

After confirming the role of C188-9 in promoting efficacy of low-dose DAC in pancreatic cancer cells, we attempted to explore its molecular mechanism. RASSF1A, located at 3p21.3, serves as a tumor suppressor determining the pathogenesis and malignancy phenotypes of various cancers; loss of RASSF1A allele is a frequent phenomenon in primary human cancer. Aberrant hypermethylation of the CpG island in the RASSF1A promoter region has been identified as the primary mechanism for the inactivation (35). It has been identified that high frequency of RASSF1A promoter hypermethylation is associated with adverse outcome of different types of malignancies (36). Based on previous studies and our results that DAC can re-express RASSF1A in a dose-dependent manner and RASSF1A was involved in DAC-mediated impairment of proliferation and EMT process, we assume that RASSF1A might be the effector that the combined treatment functions. Moreover, by detecting the methylation status of the RASSF1A promoter under combined treatment as example, it is demonstrated that C188-9 could enhance the demethylation activity of DAC, at least when targeting the RASSF1A promoter.

To clarify the mechanism how C188-9 regulates demethylation activity of DAC, Zhang et al. have reported that STAT3 and DNMT1 cooperatively induce epigenetic silencing of SHP-1 (10), which reminded us whether C188-9, a STAT3 inhibitor, promotes demethylation activity of DAC by targeting DNMT1. Consistent with a previous study, our results demonstrated that DNMT1 was regulated by C188-9, and ectopic overexpression of DNMT1 could recover the hypermethylation status of the RASSF1A promoter under combined treatment. These data indicated that C188-9 induced increase in demethylation activity of DAC, in part, was attributed to the downregulation of DNMT1.

In conclusion, we found for the first time that C188-9 treatment elevates the efficacy of DAC in managing pancreatic cancer by re-expressing RASSF1A both *in vivo* and *in vitro*. C188-9 treatment promotes the demethylation activity of DAC in reducing the methylation rate of RASSF1A by inhibiting DNMT1 expression. Our findings raise a novel regimen that improves clinical prognosis of pancreatic cancer and reveals the underlying mechanisms.

## Data Availability Statement

The datasets generated for this study are available on request to the corresponding author.

## Ethics Statement

The animal study was reviewed and approved by Animal Care and Use Committee of The First Clinical Medical School of Harbin Medical University.

## Author Contributions

RK, GS, and XL performed the cell experiment and wrote the materials and methods part. LW, LL, and YL executed the orthotopic tumor model experiment. XL, FW, PX, and SY aided in data interpretation and data analysis and wrote the introduction and results part. BS and JH participated in designing the study, instructing experiment, and writing and revising manuscript. All authors discussed, reviewed, and approved the final version of the manuscript to be published.

## Conflict of Interest

The authors declare that the research was conducted in the absence of any commercial or financial relationships that could be construed as a potential conflict of interest.

## References

[1] Siegel RL Miller KD Jemal A Cancer statistics, 2016. CA Cancer J Clin. (2016) 66:7–30. 10.3322/caac.21332 26742998

[2] Rahib L Smith BD Aizenberg R Rosenzweig AB Fleshman JM Matrisian LM. Projecting cancer incidence and deaths to 2030: the unexpected burden of thyroid, liver, and pancreas cancers in the United States. Cancer Res. (2014) 74:2913–21. 10.1158/0008-5472.CAN-14-0155 24840647

[3] Schwartsmann G Fernandes MS Schaan MD Moschen M Gerhardt LM Di Leone L. Decitabine (5-Aza-2′-deoxycytidine; DAC) plus daunorubicin as a first line treatment in patients with acute myeloid leukemia: preliminary observations. Leukemia. (1997) 11 (Suppl. 1):S28–31. 9130689

[4] Nervi C De Marinis E Codacci-Pisanelli G. Epigenetic treatment of solid tumours: a review of clinical trials. Clin Epigenet. (2015) 7:127. 10.1186/s13148-015-0157-2 26692909 PMC4676165

[5] Nie J Liu L Li X Han W. Decitabine, a new star in epigenetic therapy: the clinical application and biological mechanism in solid tumors. Cancer Lett. (2014) 354:12–20. 10.1016/j.canlet.2014.08.010 25130173

[6] Hackanson B Daskalakis M. Decitabine. Recent Results Cancer Res. (2014) 201:269–97. 10.1007/978-3-642-54490-3_18 24756800

[7] Yu H Jove R. The STATs of cancer–new molecular targets come of age. Nat Rev Cancer. (2004) 4:97–105. 10.1038/nrc1275 14964307

[8] Yu H Lee H Herrmann A Buettner R Jove R. Revisiting STAT3 signalling in cancer: new and unexpected biological functions. Nat Rev Cancer. (2014) 14:736–46. 10.1038/nrc3818 25342631

[9] Beebe JD Liu JY Zhang JT. Two decades of research in discovery of anticancer drugs targeting STAT3, how close are we? Pharmacol Ther. (2018) 191:74–91. 10.1016/j.pharmthera.2018.06.006 29933035

[10] Zhang Q Wang HY Marzec M Raghunath PN Nagasawa T Wasik MA. STAT3- and DNA methyltransferase 1-mediated epigenetic silencing of SHP-1 tyrosine phosphatase tumor suppressor gene in malignant T lymphocytes. Proc Natl Acad Sci USA. (2005) 102:6948–53. 10.1073/pnas.0501959102 15870198 PMC1100783

[11] Redell MS Ruiz MJ Alonzo TA Gerbing RB Tweardy DJ. Stat3 signaling in acute myeloid leukemia: ligand-dependent and -independent activation and induction of apoptosis by a novel small-molecule Stat3 inhibitor. Blood. (2011) 117:5701–9. 10.1182/blood-2010-04-280123 21447830 PMC3110027

[12] Jung KH Yoo W Stevenson HL Deshpande D Shen H Gagea M. Multifunctional Effects of a Small-Molecule STAT3 Inhibitor on NASH and Hepatocellular Carcinoma in Mice. Clin Cancer Res. (2017) 23:5537–46. 10.1158/1078-0432.CCR-16-2253 28533225 PMC5873583

[13] Di JX Zhang HY. C188-9, a small-molecule STAT3 inhibitor, exerts an antitumor effect on head and neck squamous cell carcinoma. Anticancer Drugs. (2019) 30:846–53. 10.1097/CAD.0000000000000783 30870229

[14] Lewis KM Bharadwaj U Eckols TK Kolosov M Kasembeli MM Fridley C. Small-molecule targeting of signal transducer and activator of transcription (STAT) 3 to treat non-small cell lung cancer. Lung Cancer. (2015) 90:182–90. 10.1016/j.lungcan.2015.09.014 26410177 PMC4619129

[15] Li L Chen H Gao Y Wang YW Zhang GQ Pan SH. Long noncoding RNA MALAT1 promotes aggressive pancreatic cancer proliferation and metastasis via the stimulation of autophagy. Mol Cancer Ther. (2016) 15:2232–43. 10.1158/1535-7163.MCT-16-0008 27371730

[16] Cheng ZX Sun B Wang SJ Gao Y Zhang YM Zhou HX. Nuclear factor-kappaB-dependent epithelial to mesenchymal transition induced by HIF-1alpha activation in pancreatic cancer cells under hypoxic conditions. PLoS ONE. (2011) 6:e23752. 10.1371/journal.pone.0023752 21887310 PMC3161785

[17] Hu J Li L Chen H Zhang G Liu H Kong R. MiR-361-3p regulates ERK1/2-induced EMT via DUSP2 mRNA degradation in pancreatic ductal adenocarcinoma. Cell Death Dis. (2018) 9:807. 10.1038/s41419-018-0839-8 30042387 PMC6057920

[18] Ji L Li L Qu F Zhang G Wang Y Bai X. Hydrogen sulphide exacerbates acute pancreatitis by over-activating autophagy via AMPK/mTOR pathway. J Cell Mol Med. (2016) 20:2349–61. 10.1111/jcmm.12928 27419805 PMC5134374

[19] Wang Y Wu X Zhou Y Jiang H Pan S Sun B. Piperlongumine suppresses growth and sensitizes pancreatic tumors to gemcitabine in a xenograft mouse model by modulating the NF-kappa B pathway. Cancer Prev Res. (2016) 9:234–44. 10.1158/1940-6207.CAPR-15-0306 26667450

[20] Han B Zhou H Jia G Wang Y Song Z Wang G. MAPKs and Hsc70 are critical to the protective effect of molecular hydrogen during the early phase of acute pancreatitis. FEBS J. (2016) 283:738–56. 10.1111/febs.13629 26683671

[21] Wang Y Zhou Y Jia G Han B Liu J Teng Y. Shikonin suppresses tumor growth and synergizes with gemcitabine in a pancreatic cancer xenograft model: Involvement of NF-kappaB signaling pathway. Biochem Pharmacol. (2014) 88:322–33. 10.1016/j.bcp.2014.01.041 24522113

[22] Kong R Sun B Jiang H Pan S Chen H Wang S. Downregulation of nuclear factor-kappaB p65 subunit by small interfering RNA synergizes with gemcitabine to inhibit the growth of pancreatic cancer. Cancer Lett. (2010) 291:90–8. 10.1016/j.canlet.2009.10.001 19880242

[23] Dastjerdi MN Azarnezhad A Hashemibeni B Salehi M Kazemi M Babazadeh Z An effective concentration of 5-Aza-CdR to induce cell death and apoptosis in human pancreatic cancer cell line through reactivating RASSF1A and up-regulation of bax genes. Iran J Med Sci. (2018) 43:533–40. 30214106 PMC6123548

[24] Dammann R Schagdarsurengin U Liu L Otto N Gimm O Dralle H. Frequent RASSF1A promoter hypermethylation and K-ras mutations in pancreatic carcinoma. Oncogene. (2003) 22:3806–12. 10.1038/sj.onc.1206582 12802288

[25] Byun DS Lee MG Chae KS Ryu BG Chi SG. Frequent epigenetic inactivation of RASSF1A by aberrant promoter hypermethylation in human gastric adenocarcinoma. Cancer Res. (2001) 61:7034–8. 11585730

[26] Jones PA Liang G. Rethinking how DNA methylation patterns are maintained. Nat Rev Genet. (2009) 10:805–11. 10.1038/nrg2651 19789556 PMC2848124

[27] Hanahan D Weinberg RA. The hallmarks of cancer. Cell. (2000) 100:57–70. 10.1016/S0092-8674(00)81683-9 10647931

[28] Baylin SB Ohm JE. Epigenetic gene silencing in cancer - a mechanism for early oncogenic pathway addiction? Nat Rev Cancer. (2006) 6:107–16. 10.1038/nrc1799 16491070

[29] Clark SJ Action at a distance: epigenetic silencing of large chromosomal regions in carcinogenesis. Hum Mol Genet. (2007) 16 Spec No 1:R88–95. 10.1093/hmg/ddm051 17613553

[30] Tsai HC Li H Van Neste L Cai Y Robert C Rassool FV. Transient low doses of DNA-demethylating agents exert durable antitumor effects on hematological and epithelial tumor cells. Cancer Cell. (2012) 21:430–46. 10.1016/j.ccr.2011.12.029 22439938 PMC3312044

[31] Scholz A Heinze S Detjen KM Peters M Welzel M Hauff P. Activated signal transducer and activator of transcription 3 (STAT3) supports the malignant phenotype of human pancreatic cancer. Gastroenterology. (2003) 125:891–905. 10.1016/S0016-5085(03)01064-3 12949733

[32] Lesina M Kurkowski MU Ludes K Rose-John S Treiber M Klöppel G. Stat3/Socs3 activation by IL-6 transsignaling promotes progression of pancreatic intraepithelial neoplasia and development of pancreatic cancer. Cancer Cell. (2011) 19:456–69. 10.1016/j.ccr.2011.03.009 21481788

[33] Wörmann SM Song L Ai J Diakopoulos KN Kurkowski MU Görgülü K. Loss of P53 function activates JAK2-STAT3 signaling to promote pancreatic tumor growth, stroma modification, and gemcitabine resistance in mice and is associated with patient survival. Gastroenterology. (2016) 151:180–93. 10.1053/j.gastro.2016.03.010 27003603

[34] Johnson DE O'Keefe RA Grandis JR. Targeting the IL-6/JAK/STAT3 signalling axis in cancer. Nat Rev Clin Oncol. (2018) 15:234–48. 10.1038/nrclinonc.2018.8 29405201 PMC5858971

[35] Lusher ME Lindsey JC Latif F Pearson AD Ellison DW Clifford SC. Biallelic epigenetic inactivation of the RASSF1A tumor suppressor gene in medulloblastoma development. Cancer Res. (2002) 62:5906–11. 12384556

[36] Grawenda AM O'neill E. Clinical utility of RASSF1A methylation in human malignancies. Br J Cancer. (2015) 113:372–81. 10.1038/bjc.2015.221 26158424 PMC4522630

