# Supplementary material for: Small Molecule Inhibitor C188-9 Synergistically Enhances the Demethylated Activity of Low-Dose 5-Aza-2′-Deoxycytidine Against Pancreatic Cancer
Source: Front Oncol. 2020 May 8;10:612. doi: 10.3389/fonc.2020.00612 (PMC7225308; doi:10.3389/fonc.2020.00612)
Supplement: Supplementary file 1 [file Table_1.DOCX]

**Table S1. Sequences of primers used in Real-time RT-PCR**

| Primer Name: | Sequences (5’ to 3’) |
| --- | --- |
| hsa-GAPDH-Forward | CCTCTGACTTCAACAGCGACAC |
| hsa-GAPDH-Reverse | TGGTCCAGGGGTCTTACTCC |
| hsa-RASSF1-Forward | CAAGTGCGTGCCGTGTGAGT |
| hsa-RASSF1-Reverse | GTAATGAGGGCAGAGGGGTG |
