# Supplementary material for: Small Molecule Inhibitor C188-9 Synergistically Enhances the Demethylated Activity of Low-Dose 5-Aza-2′-Deoxycytidine Against Pancreatic Cancer
Source: Front Oncol. 2020 May 8;10:612. doi: 10.3389/fonc.2020.00612 (PMC7225308; doi:10.3389/fonc.2020.00612)
Supplement: Supplementary file 2 [file Table_2.DOCX]

**Table S2. The target sequences of siRNAs used in transfection**

| Product Number | Product Name | Target Sequence |
| --- | --- | --- |
| siN05815122147-1-5 | siR-Ribo™  Negative Control | N/A |
| siG000011186A | si-human-RASSF1 | ACGGTTCTTACACAGGCTT |
